# {Bis[2-(dicyclo­hexyl­phosphino)­phen­yl]methyl­silyl-κ^3^ *P*,*Si*,*P*′}chloridopalladium(II)

**DOI:** 10.1107/S1600536808028961

**Published:** 2008-09-17

**Authors:** Yong-Hua Li, Yuan Zhang, Min-Min Zhao, Yu-Yu Yuan

**Affiliations:** a Ordered Matter Science Research Center, College of Chemistry and Chemical Engineering, Southeast University, Nanjing 211189, People’s Republic of China

## Abstract

In the title compound, [Pd(C_37_H_55_P_2_Si)Cl], the Pd atom has a distorted square-planar geometry. The two five-membered rings adopt envelope conformations, while the four cyclo­hexane rings have chair conformations. The two planar aromatic rings are oriented at a dihedral angle of 28.79 (3)°.

## Related literature

For general background, see: Moulton & Shaw (1976 ▶); Boom & Milstein (2003 ▶). For bond-length data, see: Allen *et al.* (1987 ▶). For ring puckering parameters, see: Cremer & Pople (1975 ▶).
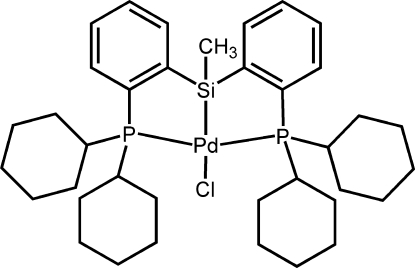


## Experimental

### 

#### Crystal data

[Pd(C_37_H_55_P_2_Si)Cl]
*M* *_r_* = 731.69
Monoclinic, 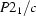

*a* = 13.079 (3) Å
*b* = 16.632 (3) Å
*c* = 17.739 (4) Å
β = 108.55 (3)°
*V* = 3658.3 (15) Å^3^
*Z* = 4
Mo *K*α radiation
μ = 0.72 mm^−1^
*T* = 153 (2) K
0.49 × 0.4 × 0.4 mm

#### Data collection

Bruker P4 diffractometer
Absorption correction: multi-scan (*CrystalClear*, Rigaku, 2005 ▶) *T*_min_ = 0.690, *T*_max_ = 0.758
26471 measured reflections
8379 independent reflections
6612 reflections with *I* > 2σ(*I*)
*R*_int_ = 0.029

#### Refinement

*R*[*F* ^2^ > 2σ(*F* ^2^)] = 0.026
*wR*(*F* ^2^) = 0.059
*S* = 0.93
8379 reflections
380 parameters
H-atom parameters constrained
Δρ_max_ = 0.83 e Å^−3^
Δρ_min_ = −0.24 e Å^−3^

### 

Data collection: *CrystalClear* (Rigaku, 2005 ▶); cell refinement: *CrystalClear*; data reduction: *CrystalStructure* (Rigaku, 2005 ▶); program(s) used to solve structure: *SHELXS97* (Sheldrick, 2008 ▶); program(s) used to refine structure: *SHELXL97* (Sheldrick, 2008 ▶); molecular graphics: *SHELXTL* (Sheldrick, 2008 ▶); software used to prepare material for publication: *SHELXTL*.

## Supplementary Material

Crystal structure: contains datablocks I, global. DOI: 10.1107/S1600536808028961/hk2519sup1.cif

Structure factors: contains datablocks I. DOI: 10.1107/S1600536808028961/hk2519Isup2.hkl

Additional supplementary materials:  crystallographic information; 3D view; checkCIF report

## Figures and Tables

**Table:** 

Pd1—P2	2.3132 (6)
Pd1—Cl1	2.4584 (6)
P1—Pd1	2.3111 (7)
Si1—Pd1	2.2829 (6)

**Table:** 

Si1—Pd1—P1	83.42 (3)
Si1—Pd1—P2	83.56 (3)
P1—Pd1—P2	160.56 (4)
Si1—Pd1—Cl1	178.18 (4)
P1—Pd1—Cl1	94.96 (3)
P2—Pd1—Cl1	98.21 (3)

**Fig. 1.**
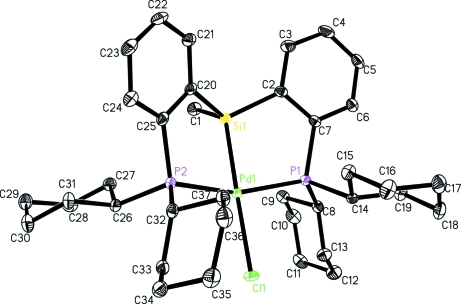
The molecular structure of the title molecule, with the atom-numbering scheme. Displacement ellipsoids are drawn at the 30% probability level. H atoms have been omitted for clarity.

## supplementary crystallographic information

### Comment

Pincer ligands incorporating two phosphine arms and a central donor site have attracted a substantial amount of interest, since the initial investigations of PCP ligands (Moulton & Shaw, 1976). Several variations of the central donor atom have been explored (Boom & Milstein, 2003). However, the "PSiP" pincer-like transition-metal complexes have rarely been reported. The title compound was obtained during our work on the phosphinoalkylsilyl complexes, and we report herein its crystal structure.

In the molecule of the title compound, (Fig. 1), the bond lengths (Allen *et al.*,  1987) and angles are generally within normal ranges. The Pd atom has a distorted square geometry (Table 1). Rings A (C2–C7) and D (C20–C25) are, of course, planar, and they are oriented at a dihedral angle of 28.79 (3)°. The six-membered rings B (C8–C13), C (C14–C19), E (C26–C31) and F (C32–C37) are not planar, having total puckering amplitudes, Q_T_, of 0.570 (3), 0.580 (3), 0.566 (3) and 0.583 (3) and chair conformations [φ = -16.92 (2)°, θ = 2.08 (3)°; φ = -100.91 (3)°, θ = 1.43 (3)°; φ = -40.78 (2)°, θ = 2.27 (3)° and φ = -155.29 (3)°, θ = 176.23 (4)°, respectively] (Cremer & Pople, 1975), while rings G (Pd1/P1/Si1/C2/C7) and H (Pd1/P2/Si1/C20/C25) adopt envelope conformations, with Si1 and Pd1 atoms displaced by -0.422 (3) and 0.694 (2) Å from the planes of the other ring atoms.

### Experimental

Dropwise addition of a solution of MeSiH(Cy_2_PC_6_H_4_)_2_ (124 mg, 0.21 mmol) in dry THF (5 ml) to a solution of [Pd(COD)Cl_2_] (43 mg, 0.21 mmol) in a mixture of THF (7 ml) and NEt_3 _(1 ml) resulted in rapid formation of a colorless precipitate. Removal of the volatiles left solid material, which after through washing left the product (yield; 130 mg, 85%). Crystals suitable for X-ray analysis were obtained by slow evaporation of a benzene (5 ml) solution of the title compound (28 mg) after 1 d.

### Refinement

H atoms were positioned geometrically, with C—H = 0.93, 0.98, 0.97 and 0.96 Å for aromatic, methine, methylene and methyl H, respectively, and constrained to ride on their parent atoms with U_iso_(H) = xU_eq_(C), where x = 1.5 for methyl H and x = 1.2 for all other H atoms.

### Crystal data

**Table:**

[Pd(C_37_H_55_P_2_Si)Cl]	*F*(000) = 1536
*M**_r_* = 731.69	*D*_x_ = 1.328 Mg m^−^^3^
Monoclinic, *P*2_1_/*c*	Mo *K*α radiation, λ = 0.71073 Å
Hall symbol: -P 2ybc	Cell parameters from 8380 reflections
*a* = 13.079 (3) Å	θ = 2.6–27.5°
*b* = 16.632 (3) Å	µ = 0.73 mm^−^^1^
*c* = 17.739 (4) Å	*T* = 153 K
β = 108.55 (3)°	Plate, colourless
*V* = 3658.3 (15) Å^3^	0.49 × 0.4 × 0.4 mm
*Z* = 4	

### Data collection

**Table:**

Bruker P4 diffractometer	8379 independent reflections
Radiation source: fine-focus sealed tube	6612 reflections with *I* > 2σ(*I*)
graphite	*R*_int_ = 0.029
Detector resolution: 13.6612 pixels mm^-1^	θ_max_ = 27.5°, θ_min_ = 1.6°
ω scans	*h* = −16→16
Absorption correction: multi-scan (CrystalClear, Rigaku, 2005)	*k* = −21→20
*T*_min_ = 0.690, *T*_max_ = 0.758	*l* = −22→23
26471 measured reflections	

### Refinement

**Table:**

Refinement on *F*^2^	Primary atom site location: structure-invariant direct methods
Least-squares matrix: full	Secondary atom site location: difference Fourier map
*R*[*F*^2^ > 2σ(*F*^2^)] = 0.026	Hydrogen site location: inferred from neighbouring sites
*wR*(*F*^2^) = 0.059	H-atom parameters constrained
*S* = 0.93	*w* = 1/[σ^2^(*F*_o_^2^) + (0.0286*P*)^2^] where *P* = (*F*_o_^2^ + 2*F*_c_^2^)/3
8379 reflections	(Δ/σ)_max_ = 0.002
380 parameters	Δρ_max_ = 0.83 e Å^−^^3^
0 restraints	Δρ_min_ = −0.24 e Å^−^^3^

### Special details

**Table:**

Geometry. All e.s.d.'s (except the e.s.d. in the dihedral angle between two l.s. planes) are estimated using the full covariance matrix. The cell e.s.d.'s are taken into account individually in the estimation of e.s.d.'s in distances, angles and torsion angles; correlations between e.s.d.'s in cell parameters are only used when they are defined by crystal symmetry. An approximate (isotropic) treatment of cell e.s.d.'s is used for estimating e.s.d.'s involving l.s. planes.
Refinement. Refinement of *F*^2^ against ALL reflections. The weighted *R*-factor *wR* and goodness of fit *S* are based on *F*^2^, conventional *R*-factors *R* are based on *F*, with *F* set to zero for negative *F*^2^. The threshold expression of *F*^2^ > σ(*F*^2^) is used only for calculating *R*-factors(gt) *etc*. and is not relevant to the choice of reflections for refinement. *R*-factors based on *F*^2^ are statistically about twice as large as those based on *F*, and *R*- factors based on ALL data will be even larger.

### Fractional atomic coordinates and isotropic or equivalent isotropic displacement parameters (Å^2^)

**Table:**

	*x*	*y*	*z*	*U*_iso_*/*U*_eq_	
Pd1	0.776955 (11)	0.199184 (8)	0.842716 (8)	0.01619 (5)	
Cl1	0.74782 (4)	0.10723 (3)	0.94246 (3)	0.02368 (11)	
P1	0.61347 (4)	0.26445 (3)	0.81739 (3)	0.01750 (11)	
P2	0.92123 (4)	0.13330 (3)	0.82162 (3)	0.01924 (11)	
Si1	0.80048 (4)	0.28746 (3)	0.75107 (3)	0.01937 (12)	
C1	0.88282 (17)	0.38076 (11)	0.79094 (12)	0.0281 (5)	
H9	0.9566	0.3659	0.8169	0.042*	
H10	0.8547	0.4070	0.8284	0.042*	
H11	0.8789	0.4167	0.7478	0.042*	
C2	0.66587 (16)	0.33238 (11)	0.69303 (11)	0.0217 (4)	
C3	0.64531 (18)	0.37785 (12)	0.62354 (12)	0.0311 (5)	
H1	0.6968	0.3798	0.5976	0.037*	
C4	0.55014 (19)	0.42001 (12)	0.59254 (13)	0.0355 (5)	
H2	0.5373	0.4483	0.5452	0.043*	
C5	0.47352 (17)	0.42045 (11)	0.63167 (12)	0.0304 (5)	
H3	0.4108	0.4506	0.6119	0.037*	
C6	0.49142 (16)	0.37573 (11)	0.70008 (11)	0.0242 (4)	
H4	0.4403	0.3758	0.7264	0.029*	
C7	0.58586 (15)	0.33015 (10)	0.73039 (11)	0.0197 (4)	
C8	0.58284 (16)	0.32556 (11)	0.89412 (11)	0.0220 (4)	
H52	0.5141	0.3530	0.8691	0.026*	
C9	0.66938 (17)	0.38988 (11)	0.92582 (12)	0.0264 (5)	
H12	0.6735	0.4236	0.8823	0.032*	
H13	0.7390	0.3643	0.9490	0.032*	
C10	0.64322 (19)	0.44220 (12)	0.98907 (13)	0.0353 (5)	
H14	0.7014	0.4802	1.0109	0.042*	
H15	0.5779	0.4726	0.9642	0.042*	
C11	0.62822 (18)	0.39211 (12)	1.05543 (12)	0.0336 (5)	
H16	0.6060	0.4265	1.0916	0.040*	
H17	0.6964	0.3674	1.0850	0.040*	
C12	0.54384 (19)	0.32688 (13)	1.02360 (13)	0.0355 (5)	
H18	0.4739	0.3517	0.9997	0.043*	
H19	0.5397	0.2934	1.0673	0.043*	
C13	0.57070 (18)	0.27452 (12)	0.96181 (12)	0.0287 (5)	
H20	0.6373	0.2456	0.9868	0.034*	
H21	0.5138	0.2354	0.9409	0.034*	
C14	0.50756 (15)	0.18646 (10)	0.78938 (11)	0.0189 (4)	
H53	0.5210	0.1499	0.8348	0.023*	
C15	0.52037 (16)	0.13733 (12)	0.72025 (12)	0.0273 (5)	
H22	0.5090	0.1720	0.6743	0.033*	
H23	0.5933	0.1165	0.7346	0.033*	
C16	0.44090 (18)	0.06758 (12)	0.69850 (14)	0.0363 (5)	
H24	0.4487	0.0396	0.6527	0.044*	
H25	0.4570	0.0299	0.7424	0.044*	
C17	0.32561 (17)	0.09697 (12)	0.67967 (14)	0.0359 (5)	
H26	0.3070	0.1307	0.6326	0.043*	
H27	0.2769	0.0513	0.6687	0.043*	
C18	0.31299 (17)	0.14426 (12)	0.74885 (14)	0.0336 (5)	
H28	0.3264	0.1094	0.7948	0.040*	
H29	0.2397	0.1642	0.7355	0.040*	
C19	0.39173 (16)	0.21510 (11)	0.76928 (13)	0.0289 (5)	
H30	0.3754	0.2517	0.7244	0.035*	
H31	0.3830	0.2442	0.8143	0.035*	
C20	0.87194 (15)	0.23153 (11)	0.68936 (11)	0.0206 (4)	
C21	0.87080 (16)	0.25321 (12)	0.61293 (12)	0.0256 (4)	
H5	0.8312	0.2978	0.5884	0.031*	
C22	0.92774 (18)	0.20930 (12)	0.57330 (12)	0.0306 (5)	
H6	0.9251	0.2240	0.5221	0.037*	
C23	0.98812 (18)	0.14409 (13)	0.60931 (13)	0.0327 (5)	
H7	1.0272	0.1154	0.5827	0.039*	
C24	0.99120 (17)	0.12082 (12)	0.68487 (12)	0.0288 (5)	
H8	1.0324	0.0768	0.7091	0.035*	
C25	0.93198 (15)	0.16385 (11)	0.72491 (11)	0.0216 (4)	
C26	1.05438 (15)	0.14745 (11)	0.89758 (11)	0.0230 (4)	
H54	1.0473	0.1279	0.9478	0.028*	
C27	1.08270 (16)	0.23684 (12)	0.90993 (12)	0.0281 (5)	
H32	1.0902	0.2590	0.8614	0.034*	
H33	1.0244	0.2651	0.9212	0.034*	
C28	1.18724 (17)	0.25026 (13)	0.97816 (13)	0.0363 (5)	
H34	1.2053	0.3070	0.9815	0.044*	
H35	1.1766	0.2346	1.0278	0.044*	
C29	1.27954 (18)	0.20260 (14)	0.96696 (14)	0.0410 (6)	
H36	1.3427	0.2090	1.0135	0.049*	
H37	1.2969	0.2235	0.9214	0.049*	
C30	1.25193 (17)	0.11356 (14)	0.95408 (14)	0.0399 (6)	
H38	1.2432	0.0910	1.0021	0.048*	
H39	1.3108	0.0855	0.9433	0.048*	
C31	1.14817 (16)	0.10098 (13)	0.88463 (13)	0.0330 (5)	
H40	1.1591	0.1189	0.8357	0.040*	
H41	1.1307	0.0441	0.8793	0.040*	
C32	0.90634 (15)	0.02306 (10)	0.81022 (12)	0.0215 (4)	
H55	0.9589	0.0051	0.7849	0.026*	
C33	0.93034 (16)	−0.02350 (11)	0.88812 (12)	0.0251 (4)	
H42	1.0016	−0.0096	0.9233	0.030*	
H43	0.8780	−0.0097	0.9144	0.030*	
C34	0.92479 (17)	−0.11331 (11)	0.86989 (13)	0.0304 (5)	
H44	0.9805	−0.1271	0.8468	0.037*	
H45	0.9387	−0.1432	0.9191	0.037*	
C35	0.81590 (18)	−0.13772 (12)	0.81309 (14)	0.0360 (5)	
H46	0.7610	−0.1296	0.8385	0.043*	
H47	0.8172	−0.1944	0.8006	0.043*	
C36	0.78722 (18)	−0.08883 (12)	0.73637 (13)	0.0348 (5)	
H48	0.8361	−0.1028	0.7072	0.042*	
H49	0.7146	−0.1022	0.7034	0.042*	
C37	0.79441 (16)	0.00105 (11)	0.75349 (12)	0.0272 (5)	
H50	0.7398	0.0162	0.7772	0.033*	
H51	0.7808	0.0305	0.7041	0.033*	

### Atomic displacement parameters (Å^2^)

**Table:**

	*U*^11^	*U*^22^	*U*^33^	*U*^12^	*U*^13^	*U*^23^
Pd1	0.01625 (8)	0.01725 (7)	0.01517 (8)	−0.00036 (6)	0.00515 (6)	0.00109 (6)
Cl1	0.0337 (3)	0.0213 (2)	0.0204 (2)	0.0042 (2)	0.0148 (2)	0.00410 (19)
P1	0.0186 (3)	0.0176 (2)	0.0161 (2)	0.0007 (2)	0.0053 (2)	0.0002 (2)
P2	0.0161 (3)	0.0211 (2)	0.0207 (3)	−0.0011 (2)	0.0061 (2)	0.0003 (2)
Si1	0.0205 (3)	0.0190 (3)	0.0187 (3)	−0.0035 (2)	0.0064 (2)	0.0012 (2)
C1	0.0316 (12)	0.0236 (10)	0.0297 (12)	−0.0045 (9)	0.0107 (10)	−0.0014 (9)
C2	0.0255 (11)	0.0183 (9)	0.0193 (10)	−0.0037 (8)	0.0044 (9)	0.0020 (8)
C3	0.0347 (13)	0.0292 (11)	0.0298 (12)	−0.0027 (10)	0.0109 (10)	0.0082 (9)
C4	0.0419 (14)	0.0309 (12)	0.0272 (12)	−0.0014 (10)	0.0017 (11)	0.0127 (10)
C5	0.0265 (12)	0.0231 (11)	0.0326 (12)	−0.0001 (9)	−0.0036 (10)	0.0058 (9)
C6	0.0231 (11)	0.0189 (10)	0.0267 (11)	−0.0027 (8)	0.0024 (9)	−0.0011 (8)
C7	0.0222 (11)	0.0154 (9)	0.0176 (10)	−0.0031 (8)	0.0011 (8)	−0.0011 (8)
C8	0.0214 (11)	0.0232 (10)	0.0210 (10)	0.0033 (8)	0.0061 (9)	−0.0021 (8)
C9	0.0300 (12)	0.0215 (10)	0.0256 (11)	0.0009 (9)	0.0060 (9)	−0.0022 (9)
C10	0.0425 (14)	0.0274 (11)	0.0311 (13)	0.0017 (10)	0.0050 (11)	−0.0088 (10)
C11	0.0397 (14)	0.0345 (12)	0.0229 (12)	0.0097 (10)	0.0045 (10)	−0.0087 (10)
C12	0.0416 (14)	0.0419 (12)	0.0272 (12)	0.0029 (11)	0.0168 (11)	−0.0060 (10)
C13	0.0358 (13)	0.0292 (11)	0.0238 (11)	−0.0013 (9)	0.0131 (10)	−0.0045 (9)
C14	0.0203 (10)	0.0191 (9)	0.0172 (10)	−0.0016 (8)	0.0057 (8)	0.0019 (8)
C15	0.0273 (12)	0.0290 (11)	0.0277 (12)	−0.0050 (9)	0.0117 (10)	−0.0074 (9)
C16	0.0365 (14)	0.0293 (12)	0.0452 (15)	−0.0061 (10)	0.0159 (12)	−0.0109 (10)
C17	0.0303 (13)	0.0286 (11)	0.0441 (14)	−0.0079 (10)	0.0050 (11)	−0.0037 (10)
C18	0.0215 (12)	0.0310 (12)	0.0491 (15)	−0.0013 (9)	0.0124 (11)	0.0019 (11)
C19	0.0246 (12)	0.0252 (11)	0.0373 (13)	−0.0008 (9)	0.0101 (10)	−0.0024 (9)
C20	0.0188 (10)	0.0222 (9)	0.0209 (10)	−0.0100 (8)	0.0066 (8)	−0.0022 (8)
C21	0.0254 (11)	0.0264 (11)	0.0247 (11)	−0.0108 (9)	0.0076 (9)	0.0001 (9)
C22	0.0366 (13)	0.0384 (12)	0.0207 (11)	−0.0174 (10)	0.0148 (10)	−0.0062 (10)
C23	0.0347 (13)	0.0370 (12)	0.0333 (13)	−0.0086 (10)	0.0204 (11)	−0.0107 (10)
C24	0.0279 (12)	0.0300 (11)	0.0314 (12)	−0.0028 (9)	0.0136 (10)	−0.0031 (9)
C25	0.0188 (10)	0.0242 (10)	0.0224 (11)	−0.0070 (8)	0.0073 (8)	−0.0042 (8)
C26	0.0169 (10)	0.0290 (11)	0.0223 (11)	−0.0028 (8)	0.0052 (8)	0.0017 (9)
C27	0.0244 (12)	0.0331 (11)	0.0254 (11)	−0.0080 (9)	0.0059 (9)	0.0007 (9)
C28	0.0318 (13)	0.0416 (13)	0.0305 (13)	−0.0158 (11)	0.0028 (10)	0.0012 (10)
C29	0.0215 (12)	0.0575 (15)	0.0391 (14)	−0.0117 (11)	0.0029 (10)	0.0053 (12)
C30	0.0193 (12)	0.0512 (15)	0.0452 (15)	0.0008 (10)	0.0045 (11)	0.0044 (12)
C31	0.0185 (11)	0.0411 (12)	0.0384 (13)	0.0007 (10)	0.0077 (10)	0.0019 (11)
C32	0.0179 (10)	0.0189 (9)	0.0288 (11)	0.0020 (8)	0.0090 (9)	−0.0009 (8)
C33	0.0204 (11)	0.0249 (10)	0.0303 (12)	0.0029 (8)	0.0083 (9)	0.0032 (9)
C34	0.0281 (12)	0.0252 (11)	0.0419 (14)	0.0055 (9)	0.0167 (11)	0.0069 (10)
C35	0.0350 (14)	0.0211 (11)	0.0535 (16)	−0.0028 (10)	0.0162 (12)	0.0007 (10)
C36	0.0293 (13)	0.0284 (11)	0.0436 (14)	−0.0053 (10)	0.0073 (11)	−0.0077 (10)
C37	0.0220 (11)	0.0255 (10)	0.0318 (12)	−0.0007 (9)	0.0055 (9)	−0.0004 (9)

### Geometric parameters (Å, °)

**Table:**

Pd1—P2	2.3132 (6)	C17—H26	0.9700
Pd1—Cl1	2.4584 (6)	C17—H27	0.9700
P1—Pd1	2.3111 (7)	C18—C19	1.531 (3)
P1—C8	1.8421 (18)	C18—H28	0.9700
P1—C14	1.8468 (19)	C18—H29	0.9700
P2—C26	1.847 (2)	C19—H30	0.9700
P2—C32	1.8480 (18)	C19—H31	0.9700
Si1—C2	1.888 (2)	C20—C21	1.398 (3)
Si1—C1	1.893 (2)	C20—C25	1.402 (3)
Si1—C20	1.8942 (19)	C21—C22	1.384 (3)
Si1—Pd1	2.2829 (6)	C21—H5	0.9300
C1—H9	0.9600	C22—C23	1.374 (3)
C1—H10	0.9600	C22—H6	0.9300
C1—H11	0.9600	C23—C24	1.383 (3)
C2—C3	1.397 (3)	C23—H7	0.9300
C2—C7	1.406 (2)	C24—C25	1.402 (3)
C3—C4	1.381 (3)	C24—H8	0.9300
C3—H1	0.9300	C25—P2	1.8370 (19)
C4—C5	1.389 (3)	C26—C31	1.528 (3)
C4—H2	0.9300	C26—C27	1.531 (3)
C5—C6	1.378 (3)	C26—H54	0.9800
C5—H3	0.9300	C27—C28	1.527 (3)
C6—C7	1.403 (3)	C27—H32	0.9700
C6—H4	0.9300	C27—H33	0.9700
C7—P1	1.8307 (19)	C28—C29	1.509 (3)
C8—C13	1.520 (3)	C28—H34	0.9700
C8—C9	1.529 (3)	C28—H35	0.9700
C8—H52	0.9800	C29—C30	1.524 (3)
C9—C10	1.542 (3)	C29—H36	0.9700
C9—H12	0.9700	C29—H37	0.9700
C9—H13	0.9700	C30—C31	1.530 (3)
C10—C11	1.505 (3)	C30—H38	0.9700
C10—H14	0.9700	C30—H39	0.9700
C10—H15	0.9700	C31—H40	0.9700
C11—C12	1.522 (3)	C31—H41	0.9700
C11—H16	0.9700	C32—C33	1.527 (3)
C11—H17	0.9700	C32—C37	1.534 (3)
C12—C13	1.526 (3)	C32—H55	0.9800
C12—H18	0.9700	C33—C34	1.525 (3)
C12—H19	0.9700	C33—H42	0.9700
C13—H20	0.9700	C33—H43	0.9700
C13—H21	0.9700	C34—C35	1.515 (3)
C14—C19	1.518 (3)	C34—H44	0.9700
C14—C15	1.527 (2)	C34—H45	0.9700
C14—H53	0.9800	C35—C36	1.526 (3)
C15—C16	1.523 (3)	C35—H46	0.9700
C15—H22	0.9700	C35—H47	0.9700
C15—H23	0.9700	C36—C37	1.522 (3)
C16—C17	1.518 (3)	C36—H48	0.9700
C16—H24	0.9700	C36—H49	0.9700
C16—H25	0.9700	C37—H50	0.9700
C17—C18	1.510 (3)	C37—H51	0.9700
			
Si1—Pd1—P1	83.42 (3)	C18—C17—H27	109.6
Si1—Pd1—P2	83.56 (3)	C16—C17—H27	109.6
P1—Pd1—P2	160.56 (4)	H26—C17—H27	108.1
Si1—Pd1—Cl1	178.18 (4)	C17—C18—C19	110.74 (16)
P1—Pd1—Cl1	94.96 (3)	C17—C18—H28	109.5
P2—Pd1—Cl1	98.21 (3)	C19—C18—H28	109.5
C7—P1—C8	105.23 (9)	C17—C18—H29	109.5
C7—P1—C14	105.22 (9)	C19—C18—H29	109.5
C8—P1—C14	105.40 (8)	H28—C18—H29	108.1
C7—P1—Pd1	111.50 (7)	C14—C19—C18	111.10 (16)
C8—P1—Pd1	121.38 (7)	C14—C19—H30	109.4
C14—P1—Pd1	106.91 (6)	C18—C19—H30	109.4
C25—P2—C26	108.15 (9)	C14—C19—H31	109.4
C25—P2—C32	102.06 (9)	C18—C19—H31	109.4
C26—P2—C32	104.38 (9)	H30—C19—H31	108.0
C25—P2—Pd1	109.19 (7)	C21—C20—C25	118.34 (17)
C26—P2—Pd1	116.74 (6)	C21—C20—Si1	125.34 (15)
C32—P2—Pd1	115.13 (6)	C25—C20—Si1	116.30 (13)
C2—Si1—C1	101.56 (9)	C22—C21—C20	120.99 (19)
C2—Si1—C20	115.16 (9)	C22—C21—H5	119.5
C1—Si1—C20	106.97 (8)	C20—C21—H5	119.5
C2—Si1—Pd1	109.23 (6)	C23—C22—C21	120.20 (19)
C1—Si1—Pd1	116.79 (7)	C23—C22—H6	119.9
C20—Si1—Pd1	107.38 (6)	C21—C22—H6	119.9
Si1—C1—H9	109.5	C22—C23—C24	120.45 (19)
Si1—C1—H10	109.5	C22—C23—H7	119.8
H9—C1—H10	109.5	C24—C23—H7	119.8
Si1—C1—H11	109.5	C23—C24—C25	119.8 (2)
H9—C1—H11	109.5	C23—C24—H8	120.1
H10—C1—H11	109.5	C25—C24—H8	120.1
C3—C2—C7	117.83 (18)	C20—C25—C24	120.16 (17)
C3—C2—Si1	125.42 (15)	C20—C25—P2	116.34 (13)
C7—C2—Si1	115.93 (14)	C24—C25—P2	123.40 (15)
C4—C3—C2	121.47 (19)	C31—C26—C27	110.10 (16)
C4—C3—H1	119.3	C31—C26—P2	116.21 (14)
C2—C3—H1	119.3	C27—C26—P2	110.93 (13)
C3—C4—C5	120.39 (19)	C31—C26—H54	106.3
C3—C4—H2	119.8	C27—C26—H54	106.3
C5—C4—H2	119.8	P2—C26—H54	106.3
C6—C5—C4	119.32 (19)	C28—C27—C26	111.73 (17)
C6—C5—H3	120.3	C28—C27—H32	109.3
C4—C5—H3	120.3	C26—C27—H32	109.3
C5—C6—C7	120.75 (18)	C28—C27—H33	109.3
C5—C6—H4	119.6	C26—C27—H33	109.3
C7—C6—H4	119.6	H32—C27—H33	107.9
C6—C7—C2	120.12 (17)	C29—C28—C27	111.77 (19)
C6—C7—P1	124.00 (14)	C29—C28—H34	109.3
C2—C7—P1	115.87 (14)	C27—C28—H34	109.3
C13—C8—C9	110.58 (17)	C29—C28—H35	109.3
C13—C8—P1	112.19 (13)	C27—C28—H35	109.3
C9—C8—P1	110.38 (13)	H34—C28—H35	107.9
C13—C8—H52	107.8	C28—C29—C30	111.63 (18)
C9—C8—H52	107.8	C28—C29—H36	109.3
P1—C8—H52	107.8	C30—C29—H36	109.3
C8—C9—C10	110.66 (16)	C28—C29—H37	109.3
C8—C9—H12	109.5	C30—C29—H37	109.3
C10—C9—H12	109.5	H36—C29—H37	108.0
C8—C9—H13	109.5	C29—C30—C31	111.15 (19)
C10—C9—H13	109.5	C29—C30—H38	109.4
H12—C9—H13	108.1	C31—C30—H38	109.4
C11—C10—C9	111.78 (16)	C29—C30—H39	109.4
C11—C10—H14	109.3	C31—C30—H39	109.4
C9—C10—H14	109.3	H38—C30—H39	108.0
C11—C10—H15	109.3	C26—C31—C30	111.02 (18)
C9—C10—H15	109.3	C26—C31—H40	109.4
H14—C10—H15	107.9	C30—C31—H40	109.4
C10—C11—C12	111.33 (18)	C26—C31—H41	109.4
C10—C11—H16	109.4	C30—C31—H41	109.4
C12—C11—H16	109.4	H40—C31—H41	108.0
C10—C11—H17	109.4	C33—C32—C37	110.56 (16)
C12—C11—H17	109.4	C33—C32—P2	114.83 (13)
H16—C11—H17	108.0	C37—C32—P2	110.73 (13)
C11—C12—C13	111.58 (17)	C33—C32—H55	106.7
C11—C12—H18	109.3	C37—C32—H55	106.7
C13—C12—H18	109.3	P2—C32—H55	106.7
C11—C12—H19	109.3	C34—C33—C32	108.82 (16)
C13—C12—H19	109.3	C34—C33—H42	109.9
H18—C12—H19	108.0	C32—C33—H42	109.9
C8—C13—C12	110.77 (16)	C34—C33—H43	109.9
C8—C13—H20	109.5	C32—C33—H43	109.9
C12—C13—H20	109.5	H42—C33—H43	108.3
C8—C13—H21	109.5	C35—C34—C33	112.01 (17)
C12—C13—H21	109.5	C35—C34—H44	109.2
H20—C13—H21	108.1	C33—C34—H44	109.2
C19—C14—C15	109.61 (16)	C35—C34—H45	109.2
C19—C14—P1	116.73 (13)	C33—C34—H45	109.2
C15—C14—P1	109.12 (12)	H44—C34—H45	107.9
C19—C14—H53	107.0	C34—C35—C36	111.27 (17)
C15—C14—H53	107.0	C34—C35—H46	109.4
P1—C14—H53	107.0	C36—C35—H46	109.4
C16—C15—C14	111.76 (16)	C34—C35—H47	109.4
C16—C15—H22	109.3	C36—C35—H47	109.4
C14—C15—H22	109.3	H46—C35—H47	108.0
C16—C15—H23	109.3	C37—C36—C35	111.30 (18)
C14—C15—H23	109.3	C37—C36—H48	109.4
H22—C15—H23	107.9	C35—C36—H48	109.4
C17—C16—C15	111.14 (17)	C37—C36—H49	109.4
C17—C16—H24	109.4	C35—C36—H49	109.4
C15—C16—H24	109.4	H48—C36—H49	108.0
C17—C16—H25	109.4	C36—C37—C32	110.54 (16)
C15—C16—H25	109.4	C36—C37—H50	109.5
H24—C16—H25	108.0	C32—C37—H50	109.5
C18—C17—C16	110.28 (19)	C36—C37—H51	109.5
C18—C17—H26	109.6	C32—C37—H51	109.5
C16—C17—H26	109.6	H50—C37—H51	108.1
			
Si1—Pd1—P2—C25	−21.40 (7)	C3—C4—C5—C6	−2.6 (3)
P1—Pd1—P2—C25	26.82 (9)	C4—C5—C6—C7	0.1 (3)
Cl1—Pd1—P2—C25	159.00 (6)	C5—C6—C7—C2	2.8 (3)
Si1—Pd1—P2—C26	101.61 (7)	C5—C6—C7—P1	−176.37 (15)
P1—Pd1—P2—C26	149.83 (8)	C3—C2—C7—C6	−3.1 (3)
Cl1—Pd1—P2—C26	−77.98 (7)	Si1—C2—C7—C6	167.11 (14)
Si1—Pd1—P2—C32	−135.46 (7)	C3—C2—C7—P1	176.15 (14)
P1—Pd1—P2—C32	−87.24 (9)	Si1—C2—C7—P1	−13.66 (19)
Cl1—Pd1—P2—C32	44.95 (7)	C6—C7—P1—C8	−48.12 (18)
C7—P1—Pd1—Si1	11.09 (6)	C2—C7—P1—C8	132.68 (15)
C8—P1—Pd1—Si1	−113.73 (7)	C6—C7—P1—C14	62.93 (17)
C14—P1—Pd1—Si1	125.58 (6)	C2—C7—P1—C14	−116.27 (15)
C7—P1—Pd1—P2	−37.14 (9)	C6—C7—P1—Pd1	178.46 (14)
C8—P1—Pd1—P2	−161.96 (8)	C2—C7—P1—Pd1	−0.74 (16)
C14—P1—Pd1—P2	77.35 (8)	C13—C8—C9—C10	−56.2 (2)
C7—P1—Pd1—Cl1	−169.74 (6)	P1—C8—C9—C10	179.03 (13)
C8—P1—Pd1—Cl1	65.44 (7)	C8—C9—C10—C11	55.2 (2)
C14—P1—Pd1—Cl1	−55.25 (6)	C9—C10—C11—C12	−54.3 (2)
C7—P1—C8—C13	163.86 (14)	C10—C11—C12—C13	55.0 (2)
C14—P1—C8—C13	52.94 (16)	C9—C8—C13—C12	57.1 (2)
Pd1—P1—C8—C13	−68.48 (15)	P1—C8—C13—C12	−179.20 (15)
C7—P1—C8—C9	−72.32 (15)	C11—C12—C13—C8	−56.4 (2)
C14—P1—C8—C9	176.75 (13)	C19—C14—C15—C16	−55.6 (2)
Pd1—P1—C8—C9	55.33 (15)	P1—C14—C15—C16	175.45 (15)
C7—P1—C14—C19	−61.00 (16)	C14—C15—C16—C17	56.0 (2)
C8—P1—C14—C19	49.93 (16)	C15—C16—C17—C18	−56.3 (2)
Pd1—P1—C14—C19	−179.66 (13)	C16—C17—C18—C19	57.3 (2)
C7—P1—C14—C15	63.90 (15)	C15—C14—C19—C18	56.5 (2)
C8—P1—C14—C15	174.84 (13)	P1—C14—C19—C18	−178.88 (14)
Pd1—P1—C14—C15	−54.76 (14)	C17—C18—C19—C14	−58.3 (2)
C25—P2—C26—C31	−58.80 (16)	C25—C20—C21—C22	−0.2 (3)
C32—P2—C26—C31	49.33 (16)	Si1—C20—C21—C22	178.54 (15)
Pd1—P2—C26—C31	177.66 (12)	C20—C21—C22—C23	−1.1 (3)
C25—P2—C26—C27	67.95 (14)	C21—C22—C23—C24	1.1 (3)
C32—P2—C26—C27	176.08 (13)	C22—C23—C24—C25	0.3 (3)
Pd1—P2—C26—C27	−55.59 (14)	C21—C20—C25—C24	1.6 (3)
C25—P2—C32—C33	161.75 (14)	Si1—C20—C25—C24	−177.30 (15)
C26—P2—C32—C33	49.19 (15)	C21—C20—C25—P2	−174.99 (14)
Pd1—P2—C32—C33	−80.12 (14)	Si1—C20—C25—P2	6.1 (2)
C25—P2—C32—C37	−72.17 (15)	C23—C24—C25—C20	−1.6 (3)
C26—P2—C32—C37	175.27 (13)	C23—C24—C25—P2	174.71 (15)
Pd1—P2—C32—C37	45.96 (15)	C20—C25—P2—C26	−114.32 (15)
C2—Si1—Pd1—P1	−16.64 (6)	C24—C25—P2—C26	69.22 (18)
C1—Si1—Pd1—P1	97.81 (8)	C20—C25—P2—C32	135.96 (15)
C20—Si1—Pd1—P1	−142.15 (7)	C24—C25—P2—C32	−40.50 (19)
C2—Si1—Pd1—P2	148.89 (6)	C20—C25—P2—Pd1	13.67 (16)
C1—Si1—Pd1—P2	−96.66 (8)	C24—C25—P2—Pd1	−162.79 (15)
C20—Si1—Pd1—P2	23.38 (6)	C31—C26—C27—C28	−55.6 (2)
C1—Si1—C2—C3	67.68 (18)	P2—C26—C27—C28	174.39 (14)
C20—Si1—C2—C3	−47.5 (2)	C26—C27—C28—C29	54.7 (2)
Pd1—Si1—C2—C3	−168.37 (15)	C27—C28—C29—C30	−54.0 (3)
C1—Si1—C2—C7	−101.67 (15)	C28—C29—C30—C31	55.0 (3)
C20—Si1—C2—C7	143.16 (14)	C27—C26—C31—C30	56.5 (2)
Pd1—Si1—C2—C7	22.28 (16)	P2—C26—C31—C30	−176.32 (14)
C2—Si1—C20—C21	36.01 (19)	C29—C30—C31—C26	−56.5 (2)
C1—Si1—C20—C21	−76.01 (18)	C37—C32—C33—C34	58.93 (19)
Pd1—Si1—C20—C21	157.89 (15)	P2—C32—C33—C34	−174.90 (13)
C2—Si1—C20—C25	−145.19 (14)	C32—C33—C34—C35	−57.9 (2)
C1—Si1—C20—C25	102.80 (15)	C33—C34—C35—C36	55.7 (2)
Pd1—Si1—C20—C25	−23.30 (16)	C34—C35—C36—C37	−53.8 (2)
C7—C2—C3—C4	0.6 (3)	C35—C36—C37—C32	55.2 (2)
Si1—C2—C3—C4	−168.58 (16)	C33—C32—C37—C36	−58.4 (2)
C2—C3—C4—C5	2.3 (3)	P2—C32—C37—C36	173.13 (14)
